# Spalt-like transcription factor-2 (SALL2) suppresses breast carcinogenesis by inducing apoptosis and inhibiting cell migration and invasion

**DOI:** 10.3389/fgeed.2026.1788913

**Published:** 2026-06-12

**Authors:** Sandeep Sisodiya, Payal Singh, Suryanshi Gupta, Manvi Naugain, Jyoti Rani, Asiya Khan, Sandeep Kumar, Neetu Mishra, Pranay Tanwar, Showket Hussain

**Affiliations:** 1 Cellular and Molecular Diagnostics (Molecular Biology Group), National Institute of Cancer Prevention and Research (ICMR), Noida, India; 2 Symbiosis School of Biological Sciences (SSBS), Symbiosis International (Deemed University) (SIU), Pune, India; 3 Department of Biosciences, Jamia Millia Islamia, New Delhi, India; 4 Faculty of Applied Biosciences and Biotechnology, Shoolini University of Biotechnology and Management Sciences, Solan, Himachal Pradesh, India; 5 Academy of Scientific and Innovative Research (AcSIR), Ghaziabad, India; 6 Department of Zoology, Meerut College, C.C.S. University, Meerut, India; 7 The All India Institute of Ayurveda (AIIA), New Delhi, India; 8 Lab Oncology Unit, Dr. B. R. A. Institute Rotary Cancer Hospital, All India Institute of Medical Sciences, New Delhi, India

**Keywords:** apoptosis, breast cancer, invasion, migration, spalt-like transcription factor

## Abstract

**Background:**

Spalt-like transcription factor 2 (SALL2) has emerged as a potential tumor suppressor in various malignancies; however, its role in breast cancer remains underexplored. This study examines the effect of SALL2 overexpression on breast carcinogenesis, with a particular focus on the induction of apoptosis and inhibition of cell migration and invasion, using breast cancer receptor-positive and receptor-negative cell lines.

**Methods:**

Breast cancer cell lines (MCF-7 and MDA-MB-231) were transiently transfected with a *SALL2* expression vector, and successful transfection was confirmed by real-time PCR and Western blot analysis. Functional assays performed included proliferation (MTT assay), wound healing, invasion (transwell Matrigel assay), apoptosis (flow cytometry), and assessment of mRNA expression levels of *CDKN1A* (p21), *p16*, *PMAIP1* (NOXA), *BAX*, and *MMP9* using quantitative real-time PCR.

**Results:**

We demonstrated that the transient upregulation of *SALL2* expression markedly inhibited cell migration and invasion, processes central to tumor metastasis. This effect was accompanied by a reduction in *MMP9*, a key enzyme associated with extracellular matrix degradation and metastatic potential. Furthermore, upregulated *SALL2* expression significantly promoted apoptosis, as evidenced by the upregulation of pro-apoptotic genes including *PMAIP1* (NOXA), *BAX*, *CDKN1A* (p21), and *p16*. These changes suggest that SALL2 not only impedes metastatic capacity but also enhances apoptotic signaling pathways in breast cancer cells. Importantly, the tumor-suppressive nature of SALL2 was consistent in both types of breast cancer cell lines, underscoring its broad therapeutic relevance across different breast cancer subtypes.

**Conclusion:**

Our findings indicate that *SALL2* expression inhibits cell proliferation, migration, and invasion, while inducing apoptosis in breast cancer. These findings suggest that *SALL2* may be a critical regulator of breast carcinogenesis and a potential target for therapeutics controlling tumor progression, invasion, and metastasis.

## 1 Introduction

Breast cancer is perhaps the most prevalent disease in women globally, accounting for a substantial proportion of cancer-related fatalities (Bray et al., 2024). Even with significant advancements in early detection, molecular classification, targeted therapies, and technological breakthroughs, breast cancer continues to exhibit marked heterogeneity in terms of histopathology, molecular features, clinical behavior, and treatment response (Sisodiya et al., 2024). Heterogeneity leads to therapeutic resistance, disease recurrence, and metastasis, which now demands the need for a deeper understanding of the molecular regulators governing breast cancer initiation and progression (Khan et al., 2025). In this context, transcription factors function as key regulators of essential cellular processes, including cell cycle control, apoptosis, and invasion, thereby shaping tumor behavior and serving as promising therapeutic targets (Nazir et al., 2019a).

Dysregulation of transcription factor (TF) coding genes can disrupt normal cellular checkpoints and promote oncogenic transformation (Hasan et al., 2024). Among these, members of the Spalt-like (SALL) gene family of TFs have gained increasing attention due to their involvement in embryonic development, stem cell maintenance, and cancer (Alvarez et al., 2021). The SALL family consists of four homologs (SALL1, SALL2, SALL3, and SALL4), which share conserved zinc finger domains that enable DNA binding and transcriptional regulation (Alvarez et al., 2021). While SALL4 has been widely explored and well-known as an oncogenic factor in several malignancies, including leukemia and solid tumors (Sun et al., 2022), the biological role of SALL2 appears to be more context-dependent and remains less clearly defined.

SALL2 is a zinc finger TF originally identified for its role in developmental processes, particularly in neural and ocular development (Hermosilla et al., 2017). Emerging evidence suggests that SALL2 acts as a tumor suppressor which modulates genes linked to cell cycle arrest, apoptosis, and differentiation (Escobar et al., 2015). Loss or reduced expression of *SALL2* has been reported in several cancer types, including ovarian, colorectal, and glioma, and this has been associated with enhanced proliferation and tumor aggressiveness (Hermosilla et al., 2017). Moreover, in some cancers, such as Wilms’ tumor (Alarcon-Vargas and Ronai, 2002), synovial sarcoma (Nielsen et al., 2003), oral, and testicular cancer (Alagaratnam et al., 2011), it is observed to be upregulated and functions as an oncogene (Estilo et al., 2009).

In our recent study, we have shown the downregulation of the *SALL2* gene in different breast cancer subtypes and also found it to be linked to survival (Sisodiya et al., 2026), but SALL2’s functional relevance in the molecular mechanism of breast cancer has not been completely explored, with available findings being limited and sometimes contradictory, warranting further investigation. Given the multifaceted role of TFs in coordinating cell cycle regulation, apoptosis, and invasion, SALL2 represents an attractive candidate for investigation in breast cancer. Preliminary evidence suggests that SALL2 may transcriptionally regulate genes involved in growth suppression and apoptosis; however, its downstream targets and functional impact in breast cancer cells remain poorly characterized. Importantly, breast cancer subtypes differ markedly in their molecular profiles. Estrogen receptor-positive (ER^+^) cell lines, such as MCF-7, typically exhibit less aggressive behavior, whereas TNBC cell lines, such as MDA-MB-231, display high invasiveness and metastatic potential. Studying SALL2 functions across these distinct cellular models provides valuable insight into its context-dependent function in breast cancer pathology.

The current study elucidates the effects of SALL2 overexpression in MCF-7 and MDA-MB-231 cells by employing a plasmid-based transient transfection approach. We focused on assessing the expression of key genes that regulate cell cycle (*CDKN1A* (p21) and *p16*), apoptosis (*PMAIP1* (NOXA) and *BAX*), and invasion (*MMP9*) using real-time quantitative PCR. These genes were selected based on their established roles in cancer progression and their potential regulation by transcriptional mechanisms in breast cancer. By comparing SALL2-overexpressing cells with empty vector-transfected controls, we aimed to elucidate the tumor-suppressive potential of SALL2 in breast cancer cells.

## 2 Methodology

### 2.1 Cell culture

Receptor-positive, MCF-7 (ER^+^/PR^+^) and triple-negative, MDA-MB-231 (ER^−^/PR^−^/HER2^−^) breast cancer cell lines were purchased from the repository at NCCS, Pune, India, along with STR profiling and mycoplasma testing for authentication. Cell lines were maintained and cultured in DMEM supplemented with 10% fetal bovine serum (FBS), and 1% Pen-Strep antibiotics. The cells were incubated at 37 °C, supplied with 5% CO_2_, and maintained under humid conditions.

### 2.2 Transfection

The human *SALL2* gene ORF cDNA expression plasmid, pCMV3-SALL2, and empty vector pCMV3 were procured from Sino Biologicals (Beijing, China). The plasmid was resuspended as per the manufacturer’s manual, and the SALL2 sequence was confirmed by Sanger sequencing. MCF-7 and MDA-MB-231 cells were transfected with 2 μg of pCMV3-SALL2 (expression plasmid) or pCMV3 (empty vector) using Lipofectamine 3000 (Invitrogen; Thermo Fisher Scientific) for 5 h, followed by a medium change with fresh FBS-supplemented DMEM. Assays were performed after 24 h of medium change or as stated.

### 2.3 Real-time PCR (qPCR) analysis

Total cellular RNA was isolated through the Trizol method adapted from Dubey et al. (2022). After quantifying RNA using the Qubit 3.0 Fluorometer (Thermo Fisher, United States), RNA integrity was validated by performing a quality check on a 2% ethidium bromide-stained (EtBr) agarose gel. Using a cDNA synthesis kit iScriptTM (Ref#1708841, Biorad, United States), and following the instructions stated in manufacturer’s manual, we performed cDNA synthesis with 1 μg of quality-approved RNA.

Gene-specific primers against desired targets (Table 1) were used to perform qPCR on a CFX-96 (Bio-Rad, United States) system using the SYBR Green chemistry. Here, β-actin served as a reference target for performing expression normalization, and the relative fold change calculation (for gene expression) was performed using the 2^(−ΔΔCt)^ formula.

**TABLE 1:** Primer sequence used in real-time PCR.

S. no.	Gene name	Forward primer (5′-3′)	Reverse primer (5′-3′)
1	*SALL2*	AGG​TGC​CGG​TAC​TGA​AGA​TG	CAC​TGA​CCA​GCC​AAA​ACC​TT
2	*CDKN1A* (p21)	CAG​GTC​CAC​ATG​GTC​TTC​CT	TGC​CCA​AGC​TCT​ACC​TTC​C
3	*CDKN2A* (p16)	ACC​CCG​CTT​TCG​TAG​TTT​TC	GCA​GAA​GCG​GTG​TTT​TTC​TT
4	*PMAIP1* (NOXA)	GAA​GTC​GAG​TGT​GCT​ACT​CA	CAG​AAG​AGT​TTG​GAT​ATC​AGA​T
5	*BAX*	GGG​GAC​GAA​CTG​GAC​AGT​A	CAG​TTG​AAG​TTG​CCG​TCA​GA
6	*MMP9*	CTG​GAG​ACC​TGA​GAA​CCA​A	ACT​GCT​CAA​AGC​CTC​CAC​AAG​A
7	*β-actin*	GGA​TGC​AGA​AGG​AGA​TCA​CTG	CGA​TCC​ACA​CGG​AGT​ACT​TG

### 2.4 Protein isolation and Western blotting analysis

Following transient transfection in MCF-7 and MDA-MB-231 cell lines, the medium was changed after 5 h and supplemented with fresh DMEM containing 10% FBS. After 24 h, the transfected cells were collected, and cell lysates were processed following the methods adapted from Nazir et al., (2019b), with modifications. Next, we washed the lysates two times using ice-chilled phosphate-buffered saline (PBS). We further added Pierce™ RIPA lysis buffer (Thermo Scientific, United States) along with 1% protease inhibitor cocktail (Sigma-Aldrich, United States) to our prepared lysates and extracted total cellular protein for our further analysis. We then incubated the lysates for 45 min at 4 °C, with intermittent vortexing, and subsequently clarified them by centrifuging at 19,500 × g for 0.5 h. The clear supernatant, containing soluble proteins, was carefully aspirated and collected in a fresh tube on ice. Protein concentrations were then calculated using the Qubit 3.0 fluorometer.

Equal protein concentrations (50 μg) from each sample were mixed and denatured in Laemmli buffer by heating at 95 °C for 10 min. Proteins were electrophoretically resolved in 10%–12% SDS-PAGE till the markers were visibly separated in the gel. Subsequently, proteins from the gel are transferred over methanol-charged PVDF membranes (Millipore, United States) using a semi-dry turbo transfer system (Biorad, United States). To impede non-specific binding, membranes were blocked with 5% PBSA (BSA in PBS buffer) for 45 min at room temperature (RT). Next, the membranes were treated with anti-SALL2 (Invitrogen, United States; Ref# PA5-71338; 1:500) and anti-β-actin (Abcam, United States; Ref# ab8227; 1:5000) primary antibodies (1° Ab) diluted in 2% BSA for 16 h at 4 °C. Following overnight incubation, washing was done with PBST repeatedly for three times at a medium speed of rocker shaker, followed by incubation in HRP-conjugated secondary antibodies (2° Ab) [anti-rabbit (CST; 1:5000, United States) or anti-mouse (CST; 1:5000, United States)], for 2 h at RT. Enhanced chemiluminescence (ECL) reagent (Biorad, United States) was employed to examine the target-specific protein bands on the PVDF membrane, and a chemiluminescence imaging unit (Biorad, United States) was utilized to capture the images.

### 2.5 Cell proliferation assay (MTT assay)

For evaluating cell proliferation potential, we performed MTT assay by seeding 10,000 cells in cell culture-grade 96-well plates, which were then placed and allowed to adhere properly for 12 h. Cells were then transiently transfected with the SALL2 expression and control plasmids respectively using Lipofectamine 3000. At 24 h and 48 h post-transfection, we mixed MTT solution at a concentration of 5 mg/mL and incubated the 96-well plate containing live cells at 37 °C for 3–4 h. Next, we mixed dimethyl sulfoxide (DMSO), incubated for 15 min, and measured the absorbance at 570 nm using a plate reader (SPECTROstar Nano, BMG, Germany). The percentages of cell viability and proliferation were presented as a percentage relative to the control group.

### 2.6 Scratch assay (wound healing assay)

To assess collective cell migration under *in vitro* conditions, we performed wound healing (scratch) analysis. For this, we seeded MCF-7 and MDA-MB-231 cells in culture-grade 6-well plates at 80% confluency. The cells were then transiently transfected with the SALL2 plasmid, (simultaneously with control vectors) and the medium was changed with fresh DMEM after 5 h of transfection. Post 24 h of medium change, we generated a uniform scratch using a sterile 10 µL pipette tip, and a wound was generated in the cell monolayer (Dubey et al., 2024). We gently aspirated the detached cells by washing them with PBS, and added fresh serum-free medium to minimize cell proliferation. We utilized an inverted phase-contrast microscope to capture images of the wound area immediately (0 h) and at the indicated time points (24 h and 48 h). Cell migration was expressed as the percentage of wound healed compared to the initial width of the wound, and the extent of closing of the wound was determined by measuring the scratch area (ImageJ software).

### 2.7 Invasion assay

To study the invasion potential of SALL2-transfected cells, we performed an invasion assay using BioCoat Matrigel Invasion Chambers with 8.0 µm pores (Corning, USA). Cells were transiently overexpressed with SALL2 pCMV3-SALL2 vector (along with empty pCMV3 vector as control), and after 24 h of medium change, the cells were trypsinized and resuspended in serum-free medium, and 0.05 million cells were seeded into the upper section of the Matrigel-coated inserts. We filled the lower section with DMEM containing 10% FBS to serve as a chemoattractant for the cells. After 36 h incubation in the humid CO_2_ incubator, non-invading cells from the upper section of the well were cleared with a cotton swab and fixed with 4% paraformaldehyde. Cells were stained with 1% crystal violet for visualization, and invaded cells were imaged using an inverted microscope (Radical, India). Cells in three randomly selected areas were counted at 20X original magnification to determine the number of invaded cells.

### 2.8 Apoptosis assay (Annexin-V and PI assay)

Briefly, cells were transiently transfected with the expression plasmid using Lipofectamine 3000. After 36-h of medium change, adherent and detached cells were collected in a fresh microcentrifuge tube. The cells were then washed carefully three times with cold PBS, followed by suspending in 1× binding buffer. The cells were then added with Annexin-V-FITC and propidium iodide (PI) and incubated for 10–15 min, as per the manufacturer’s protocol [Annexin-V-FITC apoptosis detection kit (BMS500FI/300, Invitrogen, USA)]. Following incubation, samples were immediately processed for flow cytometric (CytoFLEX, BD Biosciences, United States) analysis. Data were acquired for at least 10,000 events per sample and analyzed to distinguish viable (Annexin-V-negative/PI-negative), early apoptotic (Annexin-V-positive/PI-negative), late apoptotic (Annexin-V-positive/PI-positive), and necrotic (Annexin-V-negative/PI-positive) populations. The percentage of apoptotic cells was calculated and compared with the control group.

### 2.9 SALL2 genome binding analysis

To identify SALL2 promoter binding at selected gene targets, we retrieved publicly available ChIP-seq datasets from ENCODE and analyzed normalized processed data in bigwig format representing fold change over control for SALL2 ChIP-seq in human hepatocellular carcinoma; HepG2 cells [biological replicate 1 (GEO:GSM7247522) and biological replicate 2 (GEO:GSM7247523)], H3K27ac ChIP-seq in HepG2 (GEO:GSM733743), and H3K27ac ChIP-seq in human breast epithelium (female, 53 years) (GEO:GSE100978). The datasets were visualized using Integrated Genomics Viewer (IGV, UC San Diego, BROAD Institute), and binding signals were studied across the human reference genome assembly (hg38) using genome coordinates obtained from UCSC Genome Browser. Genomic regions corresponding to target genes [*PMAIP1* (NOXA), BAX, *CDKN1A* (p21), *CDKN2A* (p16), *MMP9*] were analyzed for SALL2 binding enrichment in both replicates and compared with H3K27ac signals from HepG2 and breast epithelium samples to evaluate colocalization with active chromatin regions.

### 2.10 Statistical analysis

Each experiment is performed as three independent biological replicates, and data are presented as mean ± SD or ±SEM values, unless otherwise stated. Statistical comparisons between control and transfected groups were performed using Student’s t-test. Significance levels (standard) were p < 0.05 (*), p < 0.01 (**) and p < 0.001 (***), respectively.

## 3 Results

### 3.1 Transient transfection markedly upregulated SALL2 expression

**FIGURE 1:**
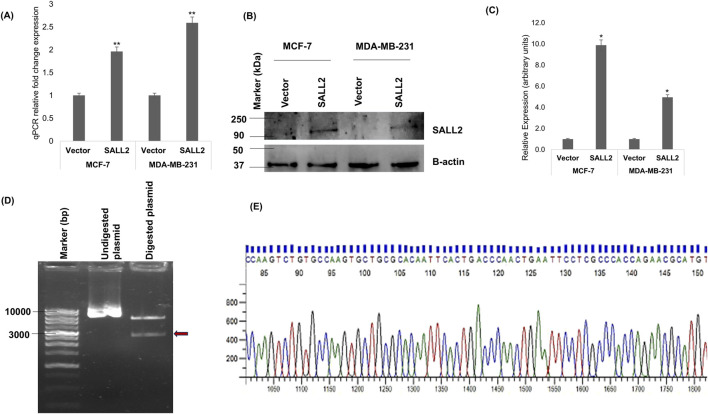
Representative image showing the successful transfection of the SALL2 plasmid. **(A)** Breast cancer cell lines (MCF-7 and MDA-MB-231) were transiently transfected with the SALL2 expression plasmid, and *SALL2* mRNA expression levels were quantified by qRT-PCR. **(B)** Western blot analysis confirmed enhanced SALL2 protein expression following transient transfection of the SALL2 plasmid in MCF-7 and MDA-MB-231 cells. **(C)** Graphical representation of densitometric analysis of Western blot for empty vector (control) and SALL expression-plasmid using ImageJ software. **(D)** To confirm the presence of the full-length SALL2 insert (2727bp), plasmid digestion was performed with KpnI and XbaI, followed by band size visualization on EtBr-stained agarose gel (Marked by an arrow). **(E)** Representative Sanger sequencing chromatogram of the recombinant plasmid verified the correct insertion and sequence integrity of the *SALL2* ORF in the expression construct. Expression levels were normalized with *β-actin*. Data are presented as mean ± SEM (n = 3). Statistical analysis was performed using Student’s t-test. Significance levels are indicated as p < 0.05 (*), and p < 0.01 (**).

We performed transient overexpression of SALL2 by transfecting MCF-7 and MDA-MB-231 cells with the full-length SALL2 coding sequence cloned into the pCMV3 expression vector. The data depicted a significant upregulation of SALL2 expression compared with control cells transfected with the empty pCMV3 vector. Quantitative RT-qPCR analysis demonstrated a marked increase in *SALL2* mRNA levels, confirming efficient plasmid-mediated overexpression (p < 0.05) in both MCF-7 and MDA-MB-231 cells (Figure 1A). Consistent with the transcriptional upregulation, Western blot analysis revealed a substantial increase in SALL2 protein levels in SALL2-transfected cells relative to controls (Figures 1B,C). These results confirm successful overexpression of the full-length *SALL2* gene at both the mRNA and protein levels. Additionally, we have also confirmed the plasmid authenticity through plasmid digestion and Sanger sequencing (Figures 1D,E).

### 3.2 Upregulation of SALL2 expression represses cell proliferation

**FIGURE 2:**
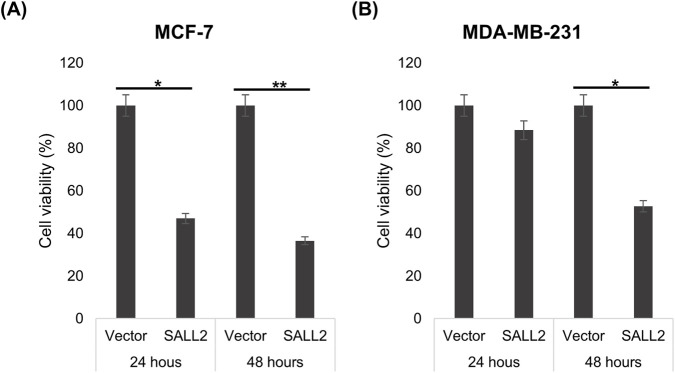
SALL2 overexpression suppresses cell proliferation in breast cancer cell lines as assessed by MTT assay. Cell proliferation/viability was evaluated using the MTT assay in **(A)** MCF-7 and **(B)** MDA-MB-231 breast cancer cell lines following transient transfection with the SALL2 expression plasmid or empty vector control. Cell viability was analyzed at 24 h and 48 h post-transfection. SALL2 overexpression resulted in a significant reduction in cell viability compared to vector-transfected controls in both cell lines, with a more pronounced effect observed at 48 h. Data are presented as the mean of three independent biological replicates. Statistical analysis was performed using Student’s t-test. Significance levels are indicated as p < 0.05 (*), and p < 0.01 (**).

To study the effect of SALL2 upregulation on breast cancer cell proliferation, MTT assay was performed in transiently transfected cells. SALL2-overexpressing MCF-7 and MDA-MB-231 cells showed a substantial decrease in live cells, compared to control (empty vector) cells, indicating suppressed proliferative capacity (Figures 2A,B). These findings demonstrate that the upregulation of SALL2 negatively regulates breast cancer cell proliferation, supporting its potential tumor-suppressive role.

### 3.3 SALL2 inhibits cell migration in breast cancer cell lines

**FIGURE 3:**
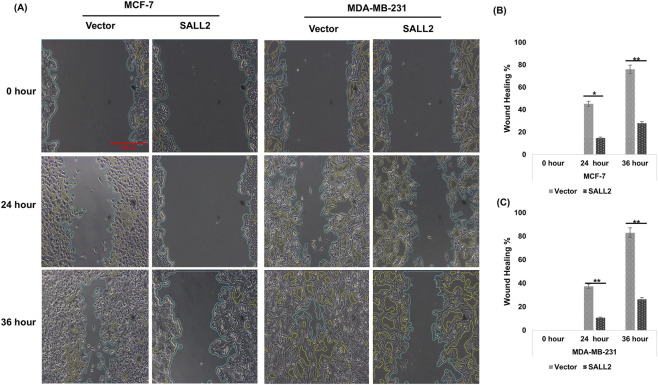
SALL2 overexpression inhibits cell migration as assessed by the wound healing assay. Cell migratory capacity was evaluated using a wound healing (scratch) assay in breast cancer cell lines (MCF-7 and MDA-MB-231) following transient transfection with the SALL2 expression plasmid or empty vector control. **(A)** Represents images of scratch taken under a phase contrast microscope at 20X original magnification, **(B,C)** are graphical representations of quantitative analysis of wound healing assay for MCF-7 and MDA-MB-231 respectively analyzed using ImageJ software. Data are presented as the mean of three independent biological replicates. Statistical analysis was performed using Student’s t-test. Significance levels are indicated as p < 0.05 (*), and p < 0.01 (**).

We next performed wound healing experiment to ascertain how SALL2 overexpression impacts the migration of MCF-7 and MDA-MB-231 cells. We generated a scratch after 24 h of medium change and analyzed the wound healing at certain time points (0 h, 24 h, and, 36 h) (Figure 3A). Our data showed a significant inhibition of wound closure in SALL2-overexpressing MCF7 and MDA-MB-231 cells compared to the vector controls at 24 h and 36 h (Figures 3B,C). Therefore, the results suggested that SALL2 has a tumor-suppressive effect by inhibiting the migratory potential of ER & PR-positive and TNBC cells.

### 3.4 Upregulation of SALL2 inhibits cell invasion

**FIGURE 4:**
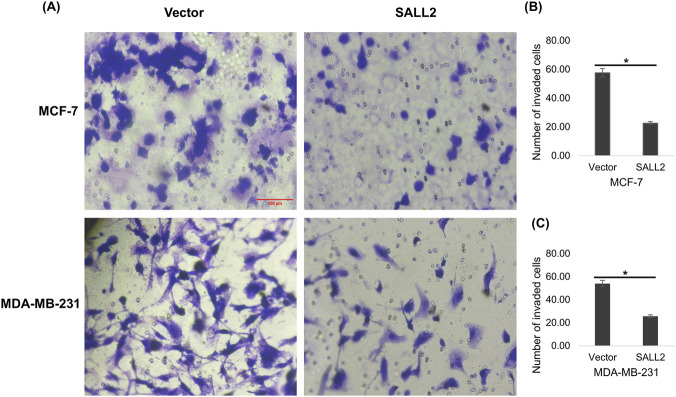
SALL2 overexpression suppresses the invasive potential of breast cancer cell lines. Cell invasion was assessed using a Matrigel-coated Transwell invasion assay in MCF-7 and MDA-MB-231 cell lines following transient transfection with the SALL2 expression plasmid or empty vector control. **(A)** Representative microscopic images of invaded cells stained with crystal violet are shown. **(B,C)** depicts the quantitative analysis for MCF-7 and MDA-MB-231 consecutively, which revealed a significant reduction in the number of invaded cells upon SALL2 overexpression compared to controls.

For assessing the effect of SALL2 on breast cancer cell invasiveness, Matrigel-based transwell invasion assay was performed. Compared with control-transfected cells, SALL2-overexpressing MCF-7 and MDA-MB-231 cells exhibited a drastic decrease in invasive capacity, as evidenced by fewer cells migrating through the Matrigel-coated membrane (Figure 4A). Quantitative analysis demonstrated a statistically significant inhibition of cell invasion in SALL2-transfected cells (p < 0.05) in MCF-7 (Figure 4B) and MDA-MB-231 cells (Figure 4C). These findings indicate that upregulation of SALL2 suppresses the invasive behavior of (ER^+^/PR^+^) and TNBC cells, demonstrating its tumor-suppressive function in both subtypes.

### 3.5 SALL2 induces apoptosis

**FIGURE 5:**
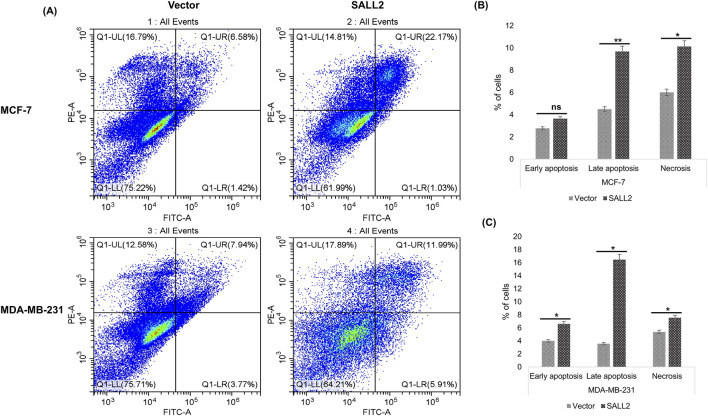
Apoptosis analysis through Annexin V-PI staining using FACS. **(A)** After transiently transfecting with the empty vector (control) and SALL2 expression plasmid, apoptosis analysis was done in breast cancer cell lines MCF-7 and MDA-MB-231, where Q1-LL for live cells (Annexin V^−^ and PI^−^), Q1-LR for early apoptosis (Annexin V^+^ and PI^−^), Q1-UR for late apoptosis (Annexin V^+^ and PI^+^), and Q1-UL was for necrosis (Annexin V^−^ and PI^+^), **(B,C)** are the graphical representations of apoptotic % in both cell lines. Statistical analysis was performed using Student’s t-test (n = 3). Significance levels are indicated as p < 0.05 (*), and p < 0.01 (**). Annexin V-FITC signals were detected in the FITC channel, while PI signals were detected in the PE (phycoerythrin) channel.

To investigate whether SALL2 affects cell death, Annexin-V-FITC and PI labelling were used to measure apoptosis in MCF-7 and MDA-MB-231 cell lines, followed by flow cytometric analysis. In comparison to control cells. Annexin V–FITC was used to detect phosphatidylserine externalization as a marker of apoptosis. Propidium iodide (PI) was used as a DNA-binding dye to assess membrane integrity and distinguish viable from non-viable cells. Quadrant distribution was defined as follows: Q1-LL (lower left) represents viable cells (Annexin V−/PI−), Q1-LR (lower right) represents early apoptotic cells (Annexin V+/PI−), Q1-UR (upper right) represents late apoptotic cells (Annexin V+/PI+), and Q1-UL (upper left) represents necrotic cells (Annexin V−/PI+) (Figure 5A). We found that SALL2 overexpression markedly increased the percentage of apoptotic cells. Quantitative analysis demonstrated a significant elevation in Annexin V-positive/PI-negative and Annexin V-positive/PI-positive levels in SALL2-overexpressing MCF-7 (Figure 5B) and MDA-MB-231 cell populations (p < 0.05) (Figure 5C). These results indicate that upregulation of SALL2 induces early and late apoptosis in breast cancer cells, contributing to its observed anti-proliferative effects.

### 3.6 SALL2 upregulation modulates expression of cell cycle and apoptotic genes

**FIGURE 6:**
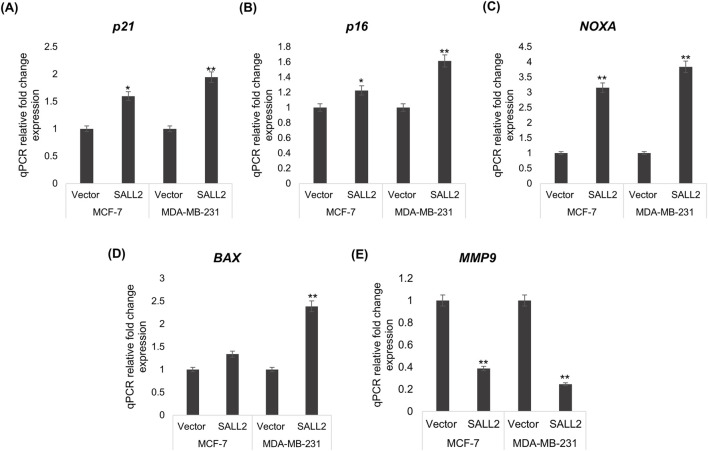
**(A–E)** Representative figure is showing SALL2 upregulates the expression of cell cycle genes, pro-apoptotic genes, and inhibits the MMP9. Breast cancer cell lines (MCF-7 and MDA-MB-231) were transiently transfected with the SALL2 expression plasmid, and relative mRNA expression of **(A)** *CDKN1A* (p21), **(B)** *CDKN2A* (p16), **(C)** *PMAIP1* (NOXA), **(D)** *BAX*, and **(E)** *MMP9* was quantified by qRT-PCR. Expression levels were normalized with *β-actin*. Statistical analysis was performed using Student’s t-test (n = 3). Significance levels are indicated as p < 0.05 (*), and p < 0.01 (**).

Since functional cellular assays showed that SALL2 significantly affected cell migration, invasion and viability, we further analyzed the target genes influenced by SALL2 TF that may drive these cellular behaviors. We observed that SALL2 overexpression led to significant modulations in the gene expression associated with cell cycle, apoptosis, and invasion, as determined by real-time quantitative PCR. In comparison to the control, SALL2-overexpressing cells showed an increase in the expression levels of the cell cycle inhibitors, *CDKN1A* (p21) and *CDKN2A* (p16), indicating enhanced cell cycle arrest (Figures 6A,B).

Subsequently, we found that the pro-apoptotic genes *PMAIP1* (NOXA) and *BAX* exhibited heightened gene expression in SALL2-overexpressing cells, indicating the activation of apoptotic pathways (Figures 6C,D). Contrastingly, *MMP9* expression was downregulated, indicating a potential inhibitory effect of SALL2 on invasion-related mechanisms (Figure 6E).

**FIGURE 7:**
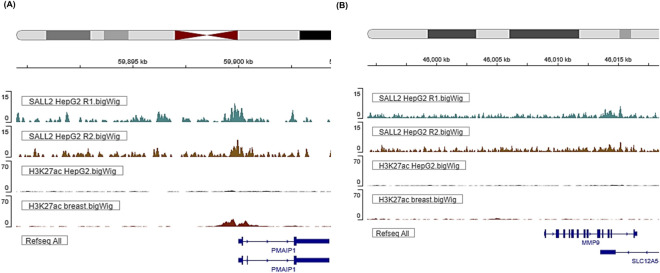
SALL2 TF binding on gene promoters. **(A)** Representative IGV tracks showing binding of SALL2 TF at *PMAIP1* (NOXA) promoter. H3K27ac peaks represent active regulatory chromatin. Blue peaks represent SALL2 HepG2 replicate 1, brown peaks represent SALL2 HepG2 replicate 2, black peaks show H3K27ac in HepG2 and red peaks represent H3K27ac breast epithelium tracks across the indicated genomic regions. **(B)** IGV tracks depicting no SALL2 enrichment at the *MMP9* genomic region.

To further investigate if SALL2-mediated gene expression changes are driven by direct transcriptional regulation by SALL2 TF, we studied the genomic occupancy of SALL2 TF using publicly available ChIP-seq datasets. As ChIP-seq data from breast tissue samples were unavailable for SALL2, we used ChIP-seq datasets generated in HepG2 cells and visualized normalized peaks in IGV. SALL2 showed robust enrichment at the *PMAIP1* (NOXA) promoter across biological replicates (Figure 7A). To assess whether the SALL2 binding is present at active regulatory regions, we simultaneously studied H3K27ac datasets derived from HepG2 and the breast epithelium of adult female. H3K27ac is a well-established marker for active promoters and enhancers, demonstrating open chromatin linked with active transcription (Konuma and Zhou, 2024; Zhang et al., 2020). Notably, SALL2 binding is strongly colocalized with H3K27ac signals in breast epithelium but not in HepG2, suggesting that SALL2 directly regulates *NOXA* transcription in breast tissues (Figure 7A). Meanwhile, the *MMP9* genomic region showed no SALL2 binding and lacked H3K27ac enrichment, suggesting the absence of active chromatin (Figure 7B). These observations further support our data showing downregulation of *MMP9* expression, suggesting that *MMP9* remains transcriptionally repressed in the presence of SALL2. We also analyzed SALL2 and H3K27ac enrichment at *p21* (*CDKN1A*), *p16* (*CDKN2A*) and *BAX* promoters. We observed no significant SALL2 binding enrichment at the *p21* promoter; however, H3K27ac signals were detected on the promoter and were highly enriched over the gene body, suggesting active promoter and enhancer-driven transcription (Supplementary Figure S1A). In contrast, SALL2 enrichment was observed on the *p16* promoter with no H3K27ac signals (Supplementary Figure S1B). This suggests that while SALL2 remains bound at the *p16* promoter, its transcriptional activation is context-dependent and may be driven by cellular conditions. Additionally, we observed SALL2 enrichment at the *BAX* promoter that was comparatively weaker than *NOXA.* The binding coincided with H3K27ac signals, indicating active transcription and SALL2-mediated regulation of BAX expression in breast cancer (Supplementary Figure S1C).

## 4 Discussion

In the current study, we demonstrated that SALL2 overexpression shows tumor-suppressive effect in breast cancer cell lines. We evidenced reduced cell proliferation, invasion, and induced apoptosis in hormone receptor-positive and TNBC models. We observed that SALL2 upregulation significantly alters key malignant phenotypes, supporting its functional relevance across molecular subtypes of breast cancer.

*SALL2* has been previously implicated in growth control, differentiation, and tumor suppression. However, its functional role in breast cancer progression remains incompletely understood. Upon exploration, one of the key findings suggests a significant reduction in cell proliferation upon *SALL2* overexpression, which was determined by the MTT assay. Since MTT reflects cellular metabolic activity and viability, the decreased signal observed in SALL2-transfected cells suggests that SALL2 negatively regulates proliferative capacity in breast cancer cells. This observation is consistent with previous reports describing *SALL2* as a growth-inhibitory factor that can modulate cell cycle progression and survival pathways by repressing cyclin D1 and E1 and restraining the G1/S cell cycle progression (Hermosilla et al., 2018). We have also observed that *SALL2* plasmid transfection showed less effect in MDA-MB-231 cell lines at 24 h, which may be attributed to intrinsic biological differences between these cell lines. MCF-7 cells are estrogen receptor-positive (ER+), hormone-responsive, and generally exhibit a more regulated and slower proliferative phenotype (Holliday and Speirs, 2011), which may make them more sensitive to early transcriptional or apoptotic changes induced by *SALL2* overexpression. MDA-MB-231 cells represent a triple-negative breast cancer (TNBC) model characterized by higher basal proliferation, genomic instability, and resistance to early apoptotic signaling. These cells often require a longer duration for phenotypic effects, such as reduced viability, to become apparent. This may explain why no significant difference is observed at 24 h, while a clear effect emerges at 48 h (Huang et al., 2020). In the current study, the antiproliferative effect observed in MCF-7 and MDA-MB-231 cells indicates that *SALL2*-mediated growth suppression may occur independently of hormone receptor status, highlighting its broader relevance in breast cancer biology.

In addition to its effects on proliferation, *SALL2* overexpression markedly inhibited cell invasion, as assessed using a Matrigel-based Transwell assay. Invasion through extracellular matrix components is a critical step in cancer metastasis, and the reduced invasive capacity of *SALL2*-overexpressing cells suggests a potential role for *SALL2* in restraining metastatic progression. Notably, the inhibitory effect was evident even in the highly invasive MDA-MB-231 cell line, underscoring the strength of *SALL2*-mediated suppression of aggressive phenotypes. These findings align with the concept that tumor-suppressing transcription factors can modulate genes involved in cell motility, extracellular matrix degradation, and epithelial-mesenchymal transition. Similarly, Miao et al. (2017) observed that *SALL2* expression may inhibit cell invasion and migration in ovarian cancer, which supports our findings that *SALL2* behaves similarly in breast cancer as well.

**FIGURE 8:**
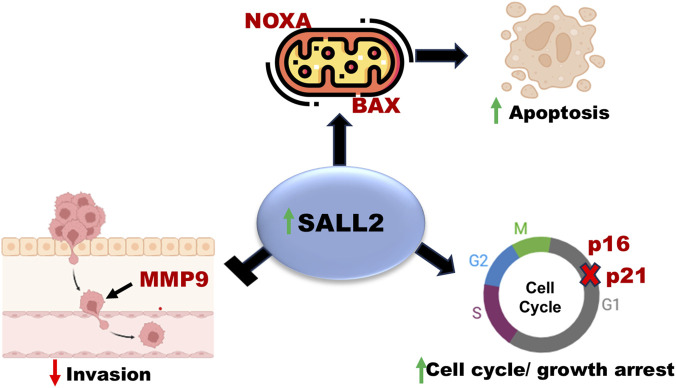
Schematic representation of the SALL2 upregulation effect on breast cancer.

Although the precise molecular mechanisms underlying SALL2-mediated suppression of proliferation and invasion were not explored in the present study, we have studied the modulation of genes associated with cell cycle (*CDKN1A* (p21) and *CDKN2A* (p16)), proapoptotic genes (*PMAIP1* (NOXA) and *BAX*), *matrix metalloproteinase-9* (MMP-9) (*MMP9*). In this study, we noticed an upregulation of *CDKN1A* (p21), *CDKN2A* (p16), *PMAIP1* (NOXA), and *BAX*, and repression of *MMP9* at the mRNA level, which suggests its role as a tumor suppressor in breast cancer (Figure 8). Additionally, we also analyzed SALL2 binding on target genes using publicly available (ENCODE) SALL2 TF ChIP-seq data sets, and observed that *SALL2* predominantly binds at *PMAIP1* (NOXA) and *BAX* promoters to regulate gene expression. These observations are consistent with the findings of Escobar et al., demonstrating that SALL2 enhances cell apoptosis under excessive cellular stress by regulating *Noxa* promoter activity and gene expression (Escobar et al., 2015). In contrast, the *CDKN1A* (p21) promoter exhibited no SALL2 binding despite the presence of active chromatin marks, while the *CDKN2A* (p16) promoter showed SALL2 enrichment with inactive chromatin. This depicts that *CDKN1A* (p21) and *p16* gene expression appear to be either indirectly regulated by SALL2 or influenced by cell-specific conditions. These interpretations partially align with the findings of Wu et al., where they have shown the direct SALL2 binding on *p16* promoter in HEK293T cells, highlighting potential cell-type-specific regulatory mechanisms (Wu et al., 2015). Notably, the *MMP9* promoter lacked detectable SALL2 binding as well as H3K27ac enrichment, indicating an absence of active chromatin at this locus. These findings are consistent with our expression data, which show downregulation of *MMP9* upon SALL2 overexpression.

*p21* (*CDKN1A*) and *p16* (*CDKN2A*) genes play a pivotal role in enforcing cell cycle checkpoints, particularly at the G1/S transition, thereby preventing uncontrolled proliferation (Abbas and Dutta, 2009; Agarwal et al., 2013). Restoration of tumor suppressive pathways that upregulate *CDKN1A* (p21) and *p16* can effectively inhibit cancer cell growth (Zhang et al., 2018). In parallel, evasion of apoptosis is another important characteristic of cancer cells. Pro-apoptotic members of the BCL-2 family, including *BAX* and *PMAIP1* (NOXA), are central mediators of the intrinsic apoptotic pathway, promoting mitochondrial outer membrane permeabilization and caspase activation. Alterations in the expression of these genes can shift the balance between cell survival and cell death, thereby influencing tumor progression and therapeutic response (Vogler et al., 2025). Additionally, Matrix metalloproteinase 9 (*MMP-9*) is a key enzyme involved in extracellular matrix remodeling and has been widely implicated in breast cancer invasion, metastasis, and poor clinical outcomes. Downregulated *MMP9* expression correlates with decreased tumor aggressiveness and metastatic potential, making it a critical molecular marker of invasive behavior (Mehner et al., 2014).

SALL2 has been reported to interact with major oncogenic signaling pathways, and its overexpression may counteract pro-tumorigenic signals that drive breast cancer progression. Further mechanistic studies will be required to delineate the downstream targets and signaling networks regulated by SALL2 in breast cancer cells. It is important to acknowledge some shortcomings of this study. Firstly, the functional analyses were primarily based on transient overexpression of SALL2, and knockdown or loss-of-function approaches were not included. While transient overexpression is a widely accepted strategy to assess gene function, complementary silencing experiments would strengthen causal interpretations. Secondly, the study focused on *in vitro* assays, further *in vivo* validation using xenograft or orthotopic models would be necessary to confirm the tumor-suppressive behavior of SALL2 in a physiological context. Finally, mechanistic insights into how SALL2 regulates proliferation and invasion remain to be elucidated and warrant future investigation.

Although the present study provides important insights into the tumor-suppressive role of SALL2 in breast cancer, several limitations should be acknowledged. This study does not directly involve genome-editing approaches; however, our findings provide a foundational basis for future investigations using epigenetic or genome-editing strategies. While we employed two distinct breast cancer cell lines representing different breast cancer subtypes, the study is limited to *in vitro* experiments, and *in vivo* validation is required to further substantiate the biological and clinical relevance of our findings. Our work primarily relies on gain-of-function (overexpression) analysis, and complementary loss-of-function approaches (e.g., gene knockdown or CRISPR-mediated knockout) would strengthen the mechanistic conclusions. Additionally, although we observed significant changes in downstream gene expression and performed bioinformatic analysis for the prediction of downstream genes, direct transcriptional regulation by SALL2 was not confirmed experimentally. Future studies incorporating techniques such as high-throughput ChIP-seq are warranted to better elucidate the transcriptional regulatory mechanisms of SALL2 in breast cancer.

In conclusion, this study provides evidence that upregulation of SALL2 suppresses key malignant phenotypes in breast cancer cells, including proliferation, invasion, and metastasis. The consistent effects observed across two distinct breast cancer cell lines support a tumor-suppressive role for SALL2 and suggest that restoration of its expression may have therapeutic relevance. These findings improve our comprehension of SALL2 function in breast cancer and lay the groundwork for future studies aimed at unraveling its molecular mechanisms and therapeutic potential for clinical significance.

## Data Availability

The original contributions presented in the study are included in the article/Supplementary Material, further inquiries can be directed to the corresponding authors.

## References

[B1] Abbas T. Dutta A. (2009). p21 in cancer: intricate networks and multiple activities. Nat. Rev. Cancer 9 (6), 400–414. 10.1038/nrc2657 19440234 PMC2722839

[B2] Agarwal P. Sandey M. DeInnocentes P. Bird R. C. (2013). Tumor suppressor gene p16/INK4A/CDKN2A-dependent regulation into and out of the cell cycle in a spontaneous canine model of breast cancer. J. Cell. Biochem. 114 (6), 1355–1363. 10.1002/jcb.24476 23238983

[B3] Alagaratnam S. Lind G. E. Kraggerud S. M. Lothe R. A. Skotheim R. I. (2011). The testicular germ cell tumour transcriptome. Int. J. Androl. 34 (4 Pt 2), e133–e150. 10.1111/j.1365-2605.2011.01169.x 21651573

[B4] Alarcon-Vargas D. Ronai Z. (2002). p53-Mdm2--the affair that never ends. Carcinogenesis 23 (4), 541–547. 10.1093/carcin/23.4.541 11960904

[B5] Alvarez C. Quiroz A. Benitez-Riquelme D. Riffo E. Castro A. F. Pincheira R. (2021). SALL proteins; common and antagonistic roles in cancer. Cancers (Basel) 13 (24), 6292. 10.3390/cancers13246292 34944911 PMC8699250

[B6] Bray F. Laversanne M. Sung H. Ferlay J. Siegel R. L. Soerjomataram I. (2024). Global cancer statistics 2022: GLOBOCAN estimates of incidence and mortality worldwide for 36 cancers in 185 countries. CA Cancer J. Clin. 74 (3), 229–263. 10.3322/caac.21834 38572751

[B7] Dubey S. Jaiswal B. Gupta A. (2022). TIP60 acts as a regulator of genes involved in filopodia formation and cell migration during wound healing. J. Biol. Chem. 298 (7), 102015. 10.1016/j.jbc.2022.102015 35525269 PMC9249863

[B8] Dubey S. Gupta H. Gupta A. (2024). Autoacetylation-mediated phase separation of TIP60 is critical for its functions. Cambridge, United Kingdom: eLife Sciences Publications, Ltd.

[B9] Escobar D. Hepp M. I. Farkas C. Campos T. Sodir N. M. Morales M. (2015). Sall2 is required for proapoptotic noxa expression and genotoxic stress-induced apoptosis by doxorubicin. Cell. Death Dis. 6 (7), e1816. 10.1038/cddis.2015.165 26181197 PMC4650718

[B10] Estilo C. L. O-charoenrat P. Talbot S. Socci N. D. Carlson D. L. Ghossein R. (2009). Oral tongue cancer gene expression profiling: identification of novel potential prognosticators by oligonucleotide microarray analysis. BMC Cancer 9, 11. 10.1186/1471-2407-9-11 19138406 PMC2649155

[B11] Hasan A. Khan N. A. Uddin S. Khan A. Q. Steinhoff M. (2024). Deregulated transcription factors in the emerging cancer hallmarks. Semin. Cancer Biol. 98, 31–50. 10.1016/j.semcancer.2023.12.001 38123029

[B12] Hermosilla V. E. Hepp M. I. Escobar D. Farkas C. Riffo E. N. Castro A. F. (2017). Developmental SALL2 transcription factor: a new player in cancer. Carcinogenesis 38 (7), 680–690. 10.1093/carcin/bgx036 28430874

[B13] Hermosilla V. E. Salgado G. Riffo E. Escobar D. Hepp M. I. Farkas C. (2018). SALL2 represses cyclins D1 and E1 expression and restrains G1/S cell cycle transition and cancer-related phenotypes. Mol. Oncol. 12 (7), 1026–1046. 10.1002/1878-0261.12308 29689621 PMC6026872

[B14] Holliday D. L. Speirs V. (2011). Choosing the right cell line for breast cancer research. Breast Cancer Res. 13 (4), 215. 10.1186/bcr2889 21884641 PMC3236329

[B15] Huang Z. Yu P. Tang J. (2020). Characterization of triple-negative breast cancer MDA-MB-231 cell spheroid model. Onco Targets Ther. 13, 5395–5405. 10.2147/OTT.S249756 32606757 PMC7295545

[B16] Khan A. Sisodiya S. Aftab M. Tanwar P. Hussain S. Gupta V. (2025). Mechanisms and therapeutic strategies for endocrine resistance in breast cancer: a comprehensive review and meta-analysis. Cancers (Basel) 17 (10), 1653. 10.3390/cancers17101653 40427153 PMC12109706

[B17] Konuma T. Zhou M. M. (2024). Distinct histone H3 lysine 27 modifications dictate different outcomes of gene transcription. J. Mol. Biol. 436 (7), 168376. 10.1016/j.jmb.2023.168376 38056822

[B18] Mehner C. Hockla A. Miller E. Ran S. Radisky D. C. Radisky E. S. (2014). Tumor cell-produced matrix metalloproteinase 9 (MMP-9) drives malignant progression and metastasis of basal-like triple negative breast cancer. Oncotarget 5 (9), 2736–2749. 10.18632/oncotarget.1932 24811362 PMC4058041

[B19] Miao F. Zhang X. Cao Y. Wang Y. Zhang X. (2017). Effect of siRNA-silencing of SALL2 gene on growth, migration and invasion of human ovarian carcinoma A2780 cells. BMC Cancer 17 (1), 838. 10.1186/s12885-017-3843-y 29228922 PMC5725831

[B20] Nazir S. U. Kumar R. Singh A. Khan A. Tanwar P. Tripathi R. (2019a). Breast cancer invasion and progression by MMP-9 through Ets-1 transcription factor. Gene 711, 143952. 10.1016/j.gene.2019.143952 31265880

[B21] Nazir S. U. Kumar R. Dil A. Rasool I. Bondhopadhyay B. Singh A. (2019b). Differential expression of Ets-1 in breast cancer among North Indian population. J. Cell. Biochem. 120 (9), 14552–14561. 10.1002/jcb.28716 31016780

[B22] Nielsen T. O. Hsu F. D. O'Connell J. X. Gilks C. B. Sorensen P. H. Linn S. (2003). Tissue microarray validation of epidermal growth factor receptor and SALL2 in synovial sarcoma with comparison to tumors of similar histology. Am. J. Pathol. 163 (4), 1449–1456. 10.1016/S0002-9440(10)63502-X 14507652 PMC1868308

[B23] Sisodiya S. Kasherwal V. Rani J. Mishra N. Kumar S. Khan A. (2024). Impact of combinatorial immunotherapies in breast cancer: a systematic review and meta-analysis. Front. Immunol. 15, 1469441. 10.3389/fimmu.2024.1469441 39478857 PMC11521824

[B24] Sisodiya S. Singh P. Joshi T. Khan A. Mishra N. Kumar S. (2026). Differential roles of SALL transcription factors in breast cancer: potential biomarkers. Comput. Biol. Med. 207, 111604. 10.1016/j.compbiomed.2026.111604 41886924

[B25] Sun B. Xu L. Bi W. Ou W. B. (2022). SALL4 oncogenic function in cancers: mechanisms and therapeutic relevance. Int. J. Mol. Sci. 23 (4), 2053. 10.3390/ijms23042053 35216168 PMC8876671

[B26] Vogler M. Braun Y. Smith V. M. Westhoff M. A. Pereira R. S. Pieper N. M. (2025). The BCL2 family: from apoptosis mechanisms to new advances in targeted therapy. Signal Transduct. Target Ther. 10 (1), 91. 10.1038/s41392-025-02176-0 40113751 PMC11926181

[B27] Wu Z. Cheng K. Shi L. Li Z. Negi H. Gao G. (2015). Sal-like protein 2 upregulates p16 expression through a proximal promoter element. Cancer Sci. 106 (3), 253–261. 10.1111/cas.12606 25580951 PMC4376433

[B28] Zhang R. Li H. Zhang S. Zhang Y. Wang N. Zhou H. (2018). RXRalpha provokes tumor suppression through p53/p21/p16 and PI3K-AKT signaling pathways during stem cell differentiation and in cancer cells. Cell. Death Dis. 9 (5), 532. 10.1038/s41419-018-0610-1 29748541 PMC5945609

[B29] Zhang T. Zhang Z. Dong Q. Xiong J. Zhu B. (2020). Histone H3K27 acetylation is dispensable for enhancer activity in mouse embryonic stem cells. Genome Biol. 21 (1), 45. 10.1186/s13059-020-01957-w 32085783 PMC7035716

