# Supplementary material for: Spalt-like transcription factor-2 (SALL2) suppresses breast carcinogenesis by inducing apoptosis and inhibiting cell migration and invasion
Source: Front Genome Ed. 2026 Jun 12;8:1788913. doi: 10.3389/fgeed.2026.1788913 (PMC13303838; doi:10.3389/fgeed.2026.1788913)
Supplement: Supplementary file 1 [file Supplementaryfile1.docx]

**Supplementary material**

**Spalt-like transcription factor-2 (SALL2) suppresses breast carcinogenesis by inducing apoptosis and inhibiting cell migration and Invasion.**

**Sandeep Sisodiya^1,2^, Payal Singh^1,3^, Suryanshi Gupta^1,4^, Manvi Naugain^1,5^, Jyoti Rani^1,6^, Asiya Khan^7^, Sandeep Kumar^1^, Neetu Mishra^2*^, Pranay Tanwar^8^, Showket Hussain^1*^**

^1^Cellular and Molecular Diagnostics (Molecular Biology Group), ICMR-National Institute of Cancer Prevention and Research, Noida, India.

^2^Symbiosis School of Biological Sciences (SSBS), Symbiosis International (Deemed University) (SIU), Pune, India.

^3^Department of Biosciences, Jamia Millia Islamia, New Delhi, India

^4^Faculty of Applied Biosciences and Biotechnology, Shoolini University of Biotechnology and Management Sciences, Himachal Pradesh, India

^5^Academy of Scientific and Innovative Research (AcSIR), Ghaziabad, India

^6^Department of Zoology, Meerut College, C.C.S. University, Meerut, India

^7^The All India Institute of Ayurveda (AIIA), New Delhi, India.

^8^Lab Oncology Unit, Dr. B.R.A. Institute Rotary Cancer Hospital, All India Institute of Medical Sciences, New Delhi, India.

***Corresponding authors**

1. **Dr. Showket Hussain**

E-mail: [showket.hussain@gov.in](mailto:showket.hussain@gov.in)

1. **Dr. Neetu Mishra**

Email: nitumishra2007@gmail.com

**Supplementary figure:**


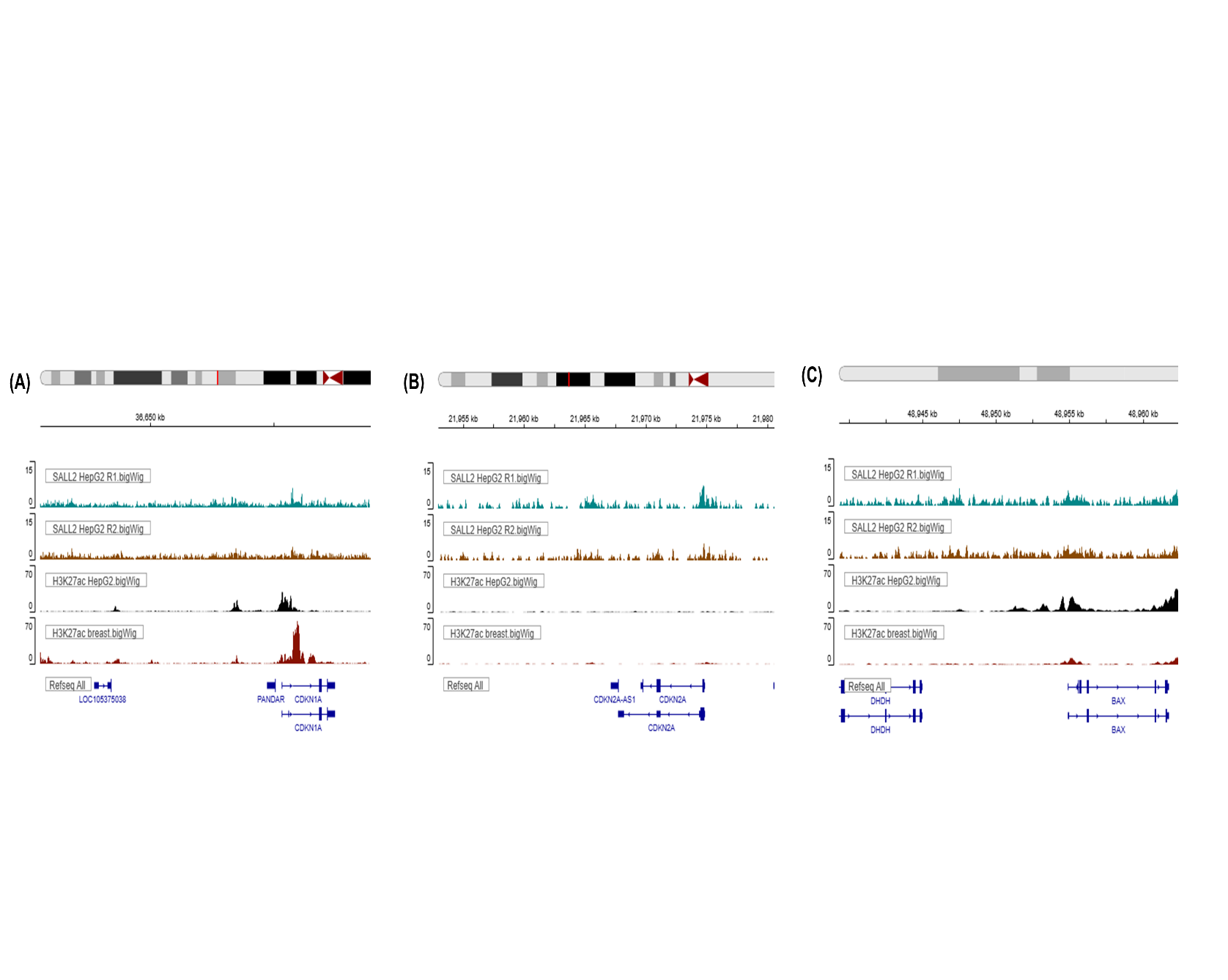


**Supplementary Figure 1**: Snapshot of IGV tracks of ChIP-seq signal for SALL2 and H3K27ac at the genomic regions of **(A)** *p21*, **(B)** *p16*, and **(C)** *BAX* gene promoters.

We analyzed *SALL2* and H3K27ac enrichment at *p21* (*CDKN1A*), *p16* (*CDKN2A*) and *BAX* promoters. We observed no *SALL2* binding at the *p21* promoter; however, H3K27ac signals were detected on the promoter and were highly enriched over the gene body, suggesting active promoter and enhancer-driven transcription. In contrast, *SALL2* enrichment was observed on the *p16* promoter with no H3K27ac signals. This suggests that while *SALL2* remains bound at the p16 promoter, its transcriptional activation is context-dependent and may be driven by cellular conditions. Similarly, we observed comparatively weak SALL2 enrichment and H3K27ac signals at the *BAX* promoter, suggesting that SALL2 may directly regulate BAX gene expression; however, we acknowledge that SALL2 enrichment efficiency on target promoters may vary across cell lines or experimental systems.
